# Synthesis of a new water-soluble hexacarboxylated tribenzotriquinacene derivative and its competitive host–guest interaction for drug delivery

**DOI:** 10.3762/bjoc.18.56

**Published:** 2022-05-12

**Authors:** Man-Ping Li, Nan Yang, Wen-Rong Xu

**Affiliations:** 1 Key Laboratory of Advanced Materials of Tropical Island Resources of Ministry of Education, Department of Chemistry, School of Science or School of Chemical Engineering and Technology, Hainan University, Haikou, 570228, China

**Keywords:** competitive substitution, drug delivery, host–guest chemistry, tribenzotriquinacene, water soluble

## Abstract

A new water-soluble hexacarboxylated tribenzotriquinacene derivative (**TBTQ-CB6**) was synthesized and used as a supramolecular drug carrier to load the model anticancer drugs dimethyl viologen (**MV**) and doxorubicin (**DOX**) via host–guest interactions. The drugs could be effectively released by spermine (**SM**), a molecule overexpressed in cancer cells, through host–guest competitive substitution since **TBTQ-CB6** has a stronger binding affinity toward **SM** than **MV** and **DOX**. The host–guest interactions of the complexes of **TBTQ-CB6** with **MV**, **DOX** and **SM** were investigated by NMR spectroscopy and fluorescence spectroscopy. The association stoichiometry of the complexes of **TBTQ-CB6** with **MV**, **DOX**, and **SM** was found to be 1:1 with association constants of *K*_a_ = (7.67 ± 0.34) × 10^4^ M^−1^, *K*_a_ = (6.81 ± 0.33) × 10^4^ M^−1^, and *K*_a_ = (5.09 ± 0.98) × 10^5^ M^−1^, respectively. The competitive substitution process was visualized by NMR titration. This novel TBTQ-based host–guest drug delivery system may have potential use in supramolecular chemotherapy.

## Introduction

Chemotherapy is considered to be one of the most effective strategies in cancer treatment [1–2]. Many types of chemotherapeutic drugs have been commonly used in clinical practice, including doxorubicin (**DOX**) [3], chlorambucil [4], oxaliplatin [5], etc. However, the use of most chemotherapeutic agents is often challenged by their poor water solubility and non-selective targeting of cancer cells, resulting in low bioavailability and systemic side effects [6]. To address these drawbacks, various approaches have been developed to improve the bioavailability of these and other drugs and to enable their targeted delivery to cancer cells [7–10]. In recent years, supramolecular chemotherapy has received considerable attention by utilizing a supramolecular strategy to decrease the cytotoxicity of anticancer drugs to normal cells while preserving their cytotoxicity against cancer cells [11]. Supramolecular systems derived from macrocycles [12–13], such as calix[*n*]arenes (CXs), cyclodextrins (CDs), cucurbiturils (CBs), and pillararenes, are of particular interest because they can act as vehicles for anticancer drugs by either self-assembling into nanocarriers [14–16] or forming host–guest complexes with anticancer drugs [17–20].

Tribenzotriquinacene (TBTQ) derivatives are a class of molecules possessing a rigid bowl-shaped structure and a *C*_3_*_v_*-symmetric skeleton [21]. Bearing similarity with macrocycles in general, they can act as versatile hosts for encapsulating guest molecules. Over the past two decades, host–guest interactions and self-assembly based on TBTQ derivatives in organic media have been well established [22–27]. Recently, the host–guest chemistry of TBTQ derivatives in aqueous phase was also investigated by us [28–29]. In particular, a water-soluble TBTQ-based hexacarboxylate (**TBTQ-C_6_**) was prepared and bound with an azobenzene-containing amphiphile (***trans*-AZO**) to produce the **TBTQ-C_6_-*trans*-AZO** supra-amphiphile by host–guest interactions in water. The supra-amphiphile was further self-assembled into photo and pH dual-responsive supramolecular vesicles that have a potential to serve as drug nanocarriers to enable controlled drug delivery [28]. These findings therefore opened up the possibility for supramolecular chemotherapy based on TBTQ derivatives. However, the use of TBTQ derivatives as hosts to directly encapsulate drugs by forming host–guest complexes has not been reported. In this paper, we report a new water-soluble derivative with an extended cavity that can associate with anticancer drugs through host–guest interactions. The resulting host–guest complexes can be considered as camouflaged anticancer drugs that may exhibit low or no cytotoxicity in normal cellular environment. Furthermore, it is hoped that the release of the drugs is regulated by competitive binding of the host to some overexpressed tumor biomarker molecules, thus restoring the antitumor bioactivity of the drugs in cancer cells.

**Scheme 1:**
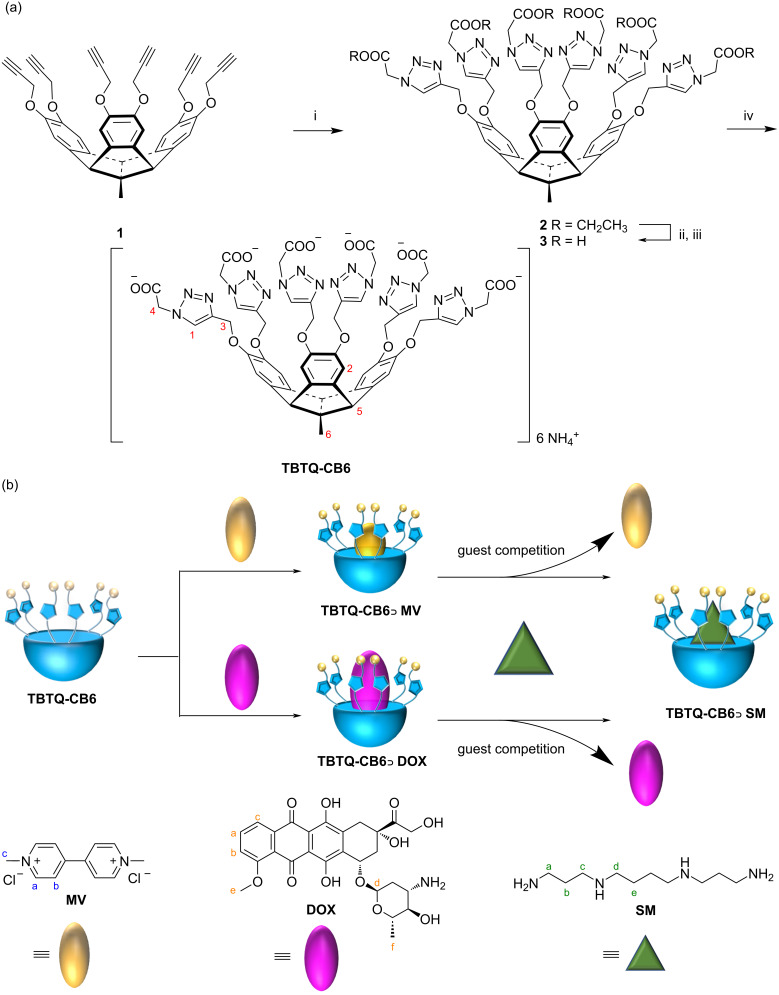
(a) Synthesis route to **TBTQ-CB6**. Conditions: (i) ethyl azidoacetate, CuSO_4_, sodium ascorbate, THF/H_2_O, 24 h, 73%; (ii) NaOH, MeOH/H_2_O, 12 h; (iii) HCl, 85% for two steps; (iv) NH_3_∙H_2_O, 6 h, 97%. (b) Schematic diagram of the construction of **TBTQ-CB6**
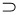
**MV** and **TBTQ-CB6**
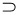
**DOX** drug delivery systems and competitive release and replacement of **MV** and **DOX** by **SM**.

We designed and synthesized a new water-soluble hexacarboxylated TBTQ derivative, **TBTQ-CB6** (Scheme 1), which features a larger cavity as compared to **TBTQ-C_6_** due to the introduction of triazole rings. Two anticancer drug molecules, dimethyl viologen (**MV**) with a smaller size and **DOX** with a larger size, were selected as model anticancer agents for encapsulation by **TBTQ-CB6** to form the host–guest complexes of **TBTQ-CB6**
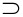
**MV** and **TBTQ-CB6**
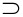
**DOX**. Spermine (**SM**), an aliphatic polyamine overexpressed in some cancer cells, was expected to exhibit a higher binding affinity to the negatively charged **TBTQ-CB6** host, because it exists mainly in a four positively charged form at physiological pH (about 7) [30–31]. Thus, **SM** was chosen as a competitive guest to control the release of **MV** and **DOX**. The structures of the target molecule **TBTQ-CB6** and its precursors were characterized by NMR spectroscopy and mass spectrometry. The host–guest chemistry of **TBTQ-CB6**
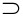
**MV**, **TBTQ-CB6**
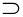
**DOX** and **TBTQ-CB6**
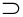
**SM** was investigated by NMR spectroscopy and fluorescence spectroscopy. The competitive binding of **TBTQ-CB6** to **SM** was revealed by NMR titration. In essence, this represents the first application of the convex-concave TBTQ motif for potential use in drug delivery systems.

## Results and Discussion

**Synthesis and characterization of host TBTQ-CB6.** The host **TBTQ-CB6** was synthesized starting from the known TBTQ-based hexakis(propargyl ether) **1** [29] (Scheme 1a). Through the CuAAC reaction with ethyl azidoacetate under Cu(I) catalysis, the TBTQ-based hexakis(ethyl acetate) compound **2** was obtained in 73% yield. Subsequent hydrolysis with sodium hydroxide followed by acidification with hydrochloric acid gave the precursor hexakis(carboxylic acid) compound **3** in 85% yield in two steps. Finally, the desired hexacarboxylated TBTQ derivative **TBTQ-CB6** was successfully obtained by reaction with ammonium hydroxide in 97% yield and found to have good water solubility. All synthesized compounds were fully characterized by ^1^H and ^13^C NMR spectroscopy and mass spectrometry (see Supporting Information File 1) and the data were found to be consistent with the proposed structures.

**Figure 1:**
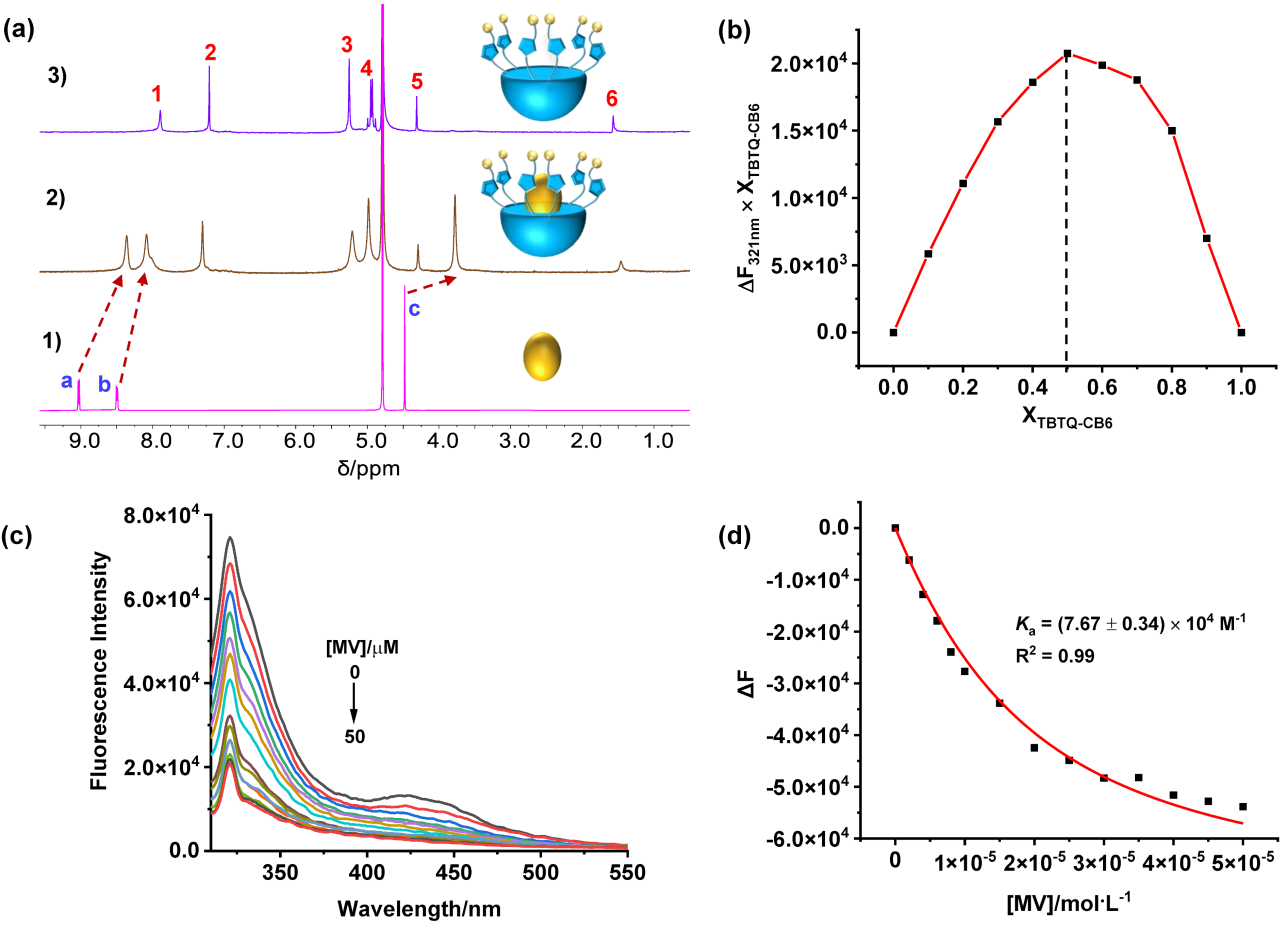
(a) ^1^H NMR spectra (400 MHz, D_2_O, 25 °C) of (1) **MV**, (2) **TBTQ-CB6** and **MV**, (3) **TBTQ-CB6** with [**TBTQ-CB6**] = [**MV**] = 3 mM in each case. (b) Job plot curve of **TBTQ-CB6** and **MV** by plotting fluorescence changes at 321 nm against the molar fraction of **TBTQ-CB6** (X**_TBTQ-CB6_**). (c) Fluorescence titration spectra of **TBTQ-CB6** (10 μM) with varying concentrations of **MV** in water (λ_ex_ = 290 nm). (d) Plot of Δ*F* vs [**MV**] at 321 nm according to (c) (the solid line was obtained from the nonlinear curve fitting).

**Host–guest complexation of TBTQ-CB6 with dimethyl viologen (MV).** The host–guest complexation between **TBTQ-CB6** and **MV** was first studied by ^1^H NMR spectroscopy. According to Figure 1a, all proton signals (H_a_, H_b_, and H_c_) of **MV** showed significant upfield shifts (∆δ = −0.67, −0.42, and −0.70 ppm, respectively) in the presence of one equivalent amount of **TBTQ-CB6** due to the shielding effect of the electron-rich cavity of **TBTQ-CB6** [32]. In addition, the proton resonances became broadened caused by the complexation dynamics, indicating the successful host–guest complexation between **TBTQ-CB6** and **MV**. Subsequently, the association stoichiometry of the complex was studied by the Job plot method using fluorescence spectroscopy (Figure S10 in Supporting Information File 1). The corresponding Job plot curve (Figure 1b) showed the maximum value at a molar fraction of 0.5 for **MV**, indicating that **TBTQ-CB6** encapsulated **MV** in a 1:1 stoichiometry. To further evaluate the association affinity of **TBTQ-CB6** and **MV**, fluorescence titration experiments were conducted. As presented in Figure 1c, the emission of **TBTQ-CB6** was gradually reduced upon increasing the **MV** concentration, suggesting charge transfer from the electron-rich host to the electron-deficient guest. According to the fluorescence titration data, the association constant was determined to be *K*_a_ = (7.67 ± 0.34) × 10^4^ M^−1^ by nonlinear curve fitting [33] (Figure 1d).

**Figure 2:**
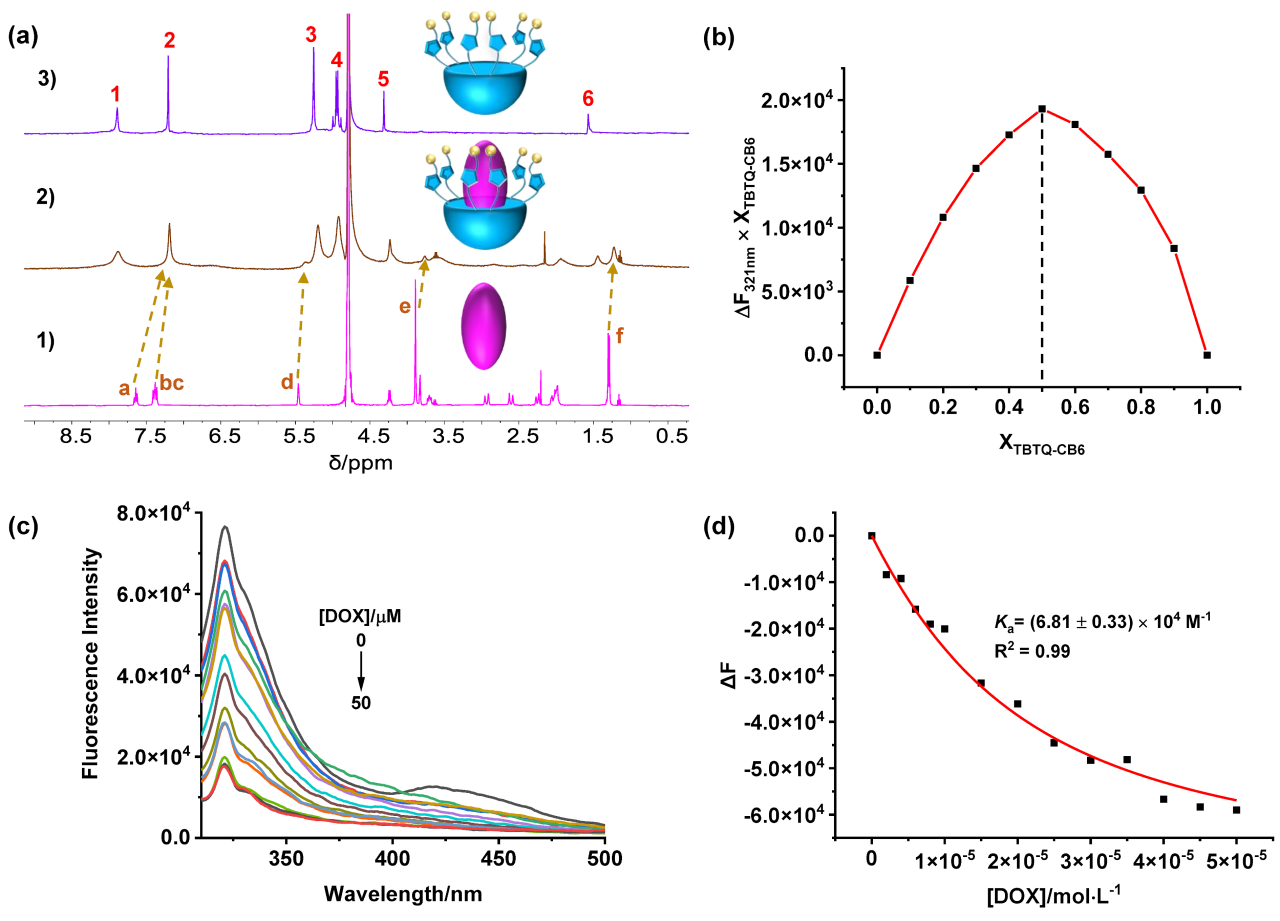
(a) ^1^H NMR spectra (400 MHz, D_2_O, 25 °C) of (1) **DOX**, (2) **TBTQ-CB6** and **DOX**, (3) **TBTQ-CB6** with [**TBTQ-CB6**] = [**DOX**] = 3 mM in each case. (b) Job plot curve of **TBTQ-CB6** and **DOX** by plotting fluorescence changes at 321 nm against the molar fraction of **TBTQ-CB6** (X**_TBTQ-CB6_**). (c) Fluorescence titration spectra of **TBTQ-CB6** (10 μM) with varying concentrations of **DOX** in water (*λ*_ex_ = 290 nm). (d) Plot of Δ*F* vs [**DOX**] at 321 nm according to (c) (the solid line was obtained from the nonlinear curve fitting).

**Host–guest complexation of TBTQ-CB6 with doxorubicin (DOX).** The host–guest complexation between **TBTQ-CB6** and **DOX** was studied by methods similar to those used for **TBTQ-CB6** and **MV**. The ^1^H NMR spectra (Figure 2a) reveal that the proton resonances of **DOX** display considerable upfield shifts upon the addition of equimolar amounts of **TBTQ-CB6**. In particular, the signals of H_a_, H_bc_, H_d_, H_e_, and H_f_ are shifted by ∆δ = −0.44, −0.18, −0.10, −0.12, and −0.07 ppm, respectively, which suggests that the electron-rich cavity of **TBTQ-CB6** exerts a shielding effect on **DOX**, i.e., **DOX** is located in the internal cavity of **TBTQ-CB6**. Furthermore, the proton signals were all significantly broadened as a result of the complexation dynamics, indicating the formation of a host–guest complex between **TBTQ-CB6** and **DOX**. Subsequently, the binding stoichiometric ratio of the complex was determined to be 1:1 by the Job plot method (Figure 2b and Figure S11 in Supporting Information File 1) through fluorescence spectrometry, and the host–guest binding constant between **TBTQ-CB6** and **DOX** was calculated to be *K*_a_ = (6.81 ± 0.33) × 10^4^ M^−1^ according to the fluorescence titration and nonlinear curve fitting (Figure 2c and 2d). The association affinity of **TBTQ-CB6**
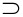
**DOX** was slightly smaller than that of **TBTQ-CB6**
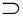
**MV**, which might result from the fact that the two positive charges of **MV** could bind more effectively to the negatively charged **TBTQ-CB6**.

**Figure 3:**
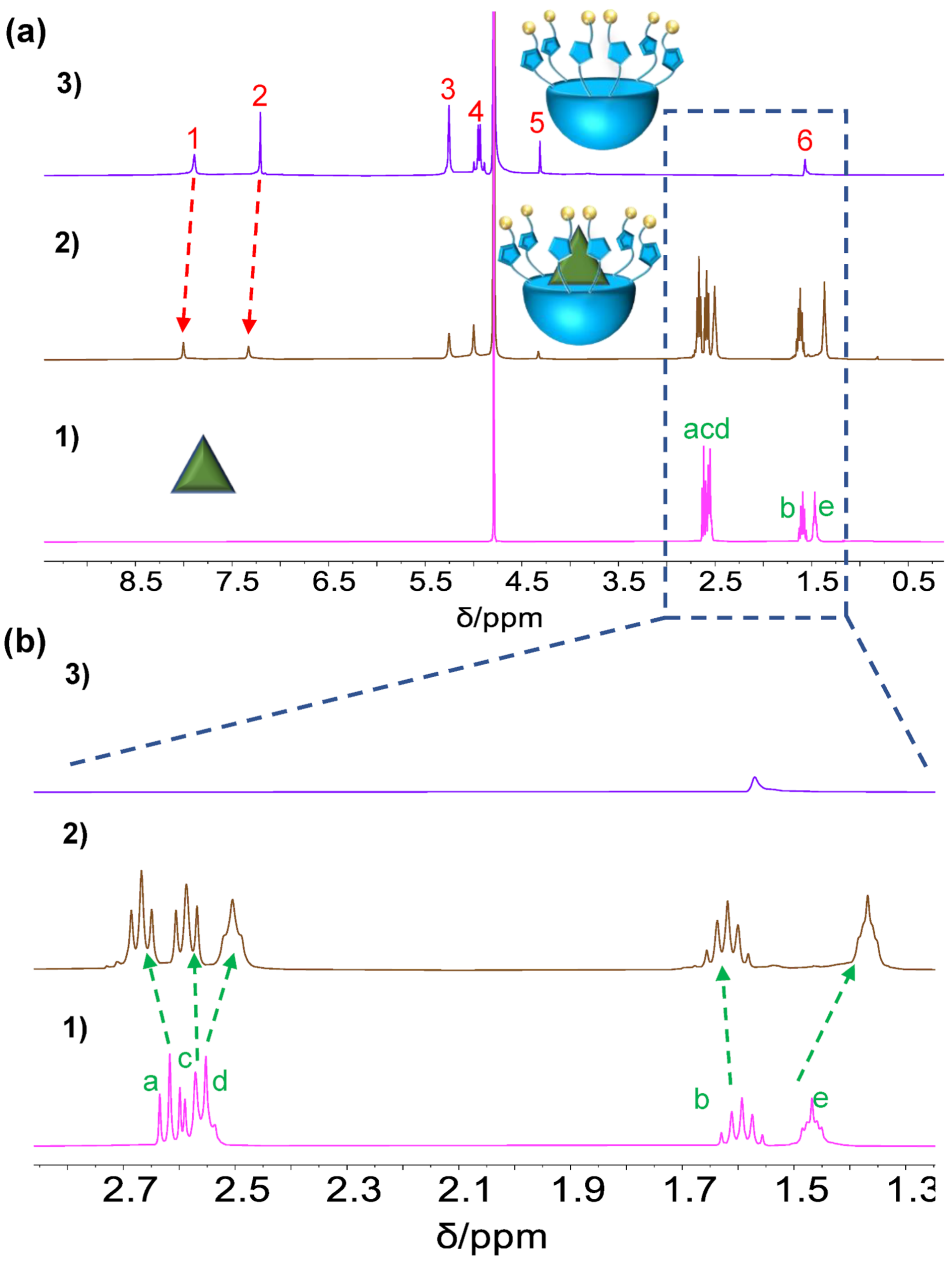
(a) ^1^H NMR spectra (400 MHz, D_2_O, 25 °C) and (b) partial magnified ^1^H NMR spectra of (1) **SM**, (2) **TBTQ-CB6** and **SM**, and (3) **TBTQ-CB6** with [**TBTQ-CB6**] = [**SM**] = 3 mM in each case.

**Figure 4:**
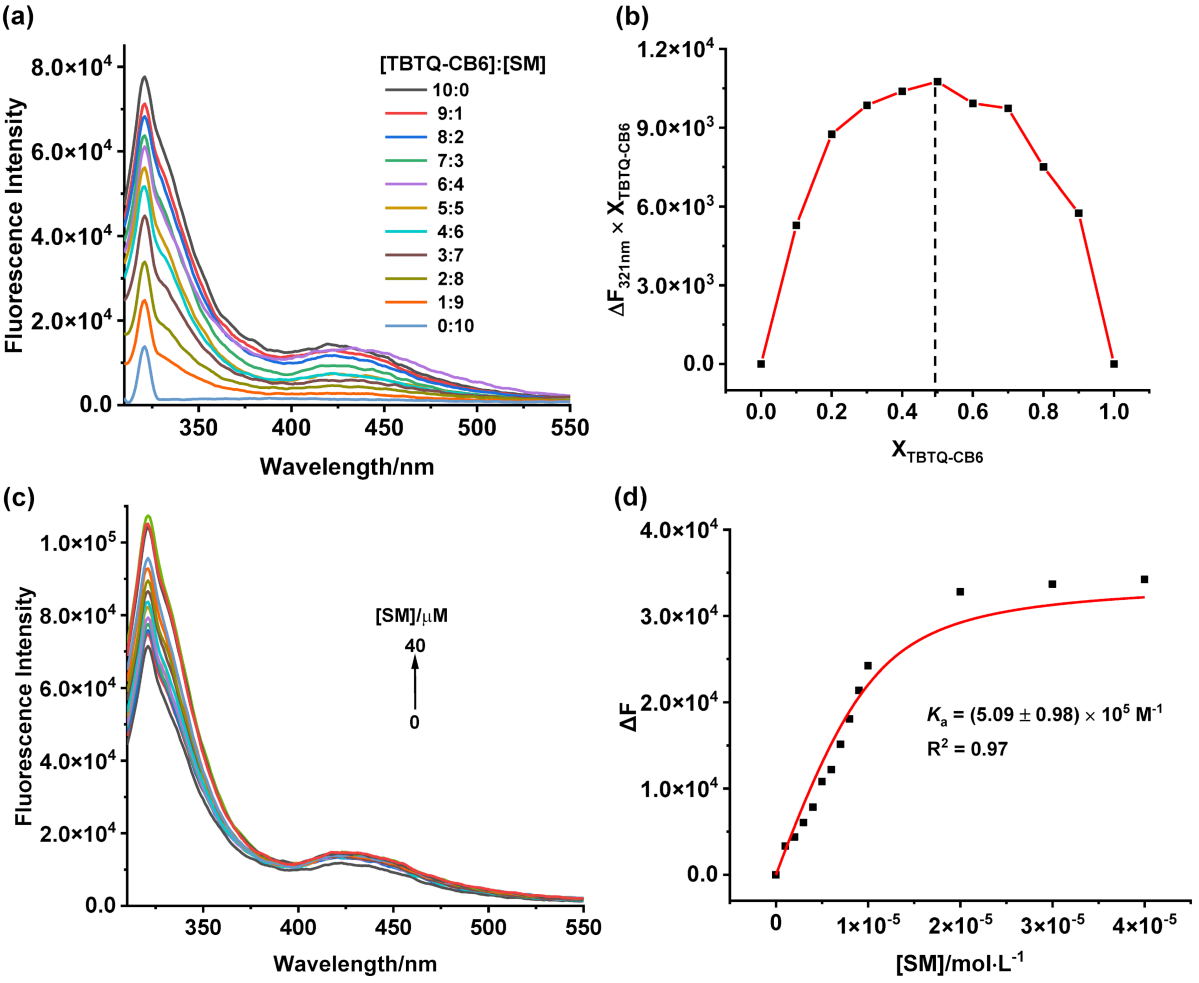
(a) Fluorescence spectra of the mixture of **TBTQ-CB6** and **SM** in different molar ratios at a constant total concentration of 10 μM. (b) Job plot of **TBTQ-CB6** and **SM** by plotting the difference in fluorescence intensity at 321 nm against the molar fraction of **TBTQ-CB6** (X_TBTQ-CB6_). (c) Fluorescence titration spectra of **TBTQ-CB6** (10 μM) with varying concentrations of **SM** in water (λ_ex_ = 290 nm). (d) Plot of Δ*F* vs [**SM**] at 321 nm according to (c) (the solid line was obtained from the nonlinear curve fitting).

**Host−guest complexation of TBTQ-CB6 with spermine (SM).** As a biomarker overexpressed in some tumor cells, **SM** was hypothesized to bind **TBTQ-CB6** more strongly than **MV** and **DOX** because of its positive charge distribution. To verify this hypothesis, the host–guest complexation between **TBTQ-CB6** and **SM** was investigated. The ^1^H NMR spectra (Figure 3) show that the proton signals (H_d_ and H_e_) located in the middle of the **SM** structure display upfield shifts (∆δ = −0.04 and −0.10 ppm, respectively) upon the addition of equimolar amounts of **TBTQ-CB6**. Furthermore, the proton resonances (H^1^ and H^2^) of **TBTQ-CB6** shifted downfield (∆δ = +0.11 and +0.12 ppm, respectively), indicating the successful binding of **SM** to **TBTQ-CB6**. The subsequent Job plot curve obtained from the fluorescence spectroscopy (Figure 4a and 4b) revealed a 1:1 stoichiometric ratio between **TBTQ-CB6** and **SM**. According to the fluorescence titration experiments and the corresponding nonlinear fitting curve (Figure 4c and 4d), the binding constant *K*_a_ for **TBTQ-CB6** and **SM** was determined to be (5.09 ± 0.98) × 10^5^ M^−1^. Compared with the binding constants determined for **TBTQ-CB6**
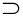
**MV** and **TBTQ-CB6**
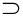
**DOX**, the higher binding constant of **TBTQ-CB6**
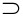
**SM** should give **SM** a competitive binding advantage to **TBTQ-CB6** and, therefore, enable the release of **MV** and **DOX** from **TBTQ-CB6**
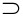
**MV** and **TBTQ-CB6**
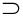
**DOX** complexes.

**Competitive replacement of MV from the TBTQ-CB6**
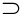
**MV complex by SM.**^1^H NMR titrations [20] were performed to characterize the competitive binding process of **TBTQ-CB6** to **SM** and the release of **MV** from the **TBTQ-CB6**
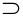
**MV** complex. Successive amounts of **SM**, ranging from 0.25 to 2.00 equivalents, were quantitatively dosed into aqueous solutions of **TBTQ-CB6**
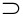
**MV** (3.0 mM). As shown in Figure 5, the proton resonances associated with **MV** (H_a_, H_b_, and H_c_ highlighted in blue) in complex **TBTQ-CB6**
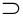
**MV** shifted gradually back downfield and became sharper with the increasing concentration of **SM**, suggesting a gradual destruction of the **TBTQ-CB6**
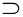
**MV** host–guest complex. It should be noted that the resonances of **MV** did not change significantly after the addition of 1.00 equivalent of **SM**, indicating that most of the **MV** had been released and gradually reached equilibrium. In addition, the proton signals (green H_d_ and H_e_) of **SM** were located upfield in comparison with the free **SM** (see Figure 3) and the **TBTQ-CB6**
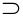
**SM** complex, which might be because **SM** was embedded in the cavity of the host. These results suggested that **MV** could be effectively released from the cavity of **TBTQ-CB6** by the competitive host–guest interaction of **SM**, since the binding affinity between **TBTQ-CB6** and **SM** was about seven times higher than that between **TBTQ-CB6** and **MV**.

**Figure 5:**
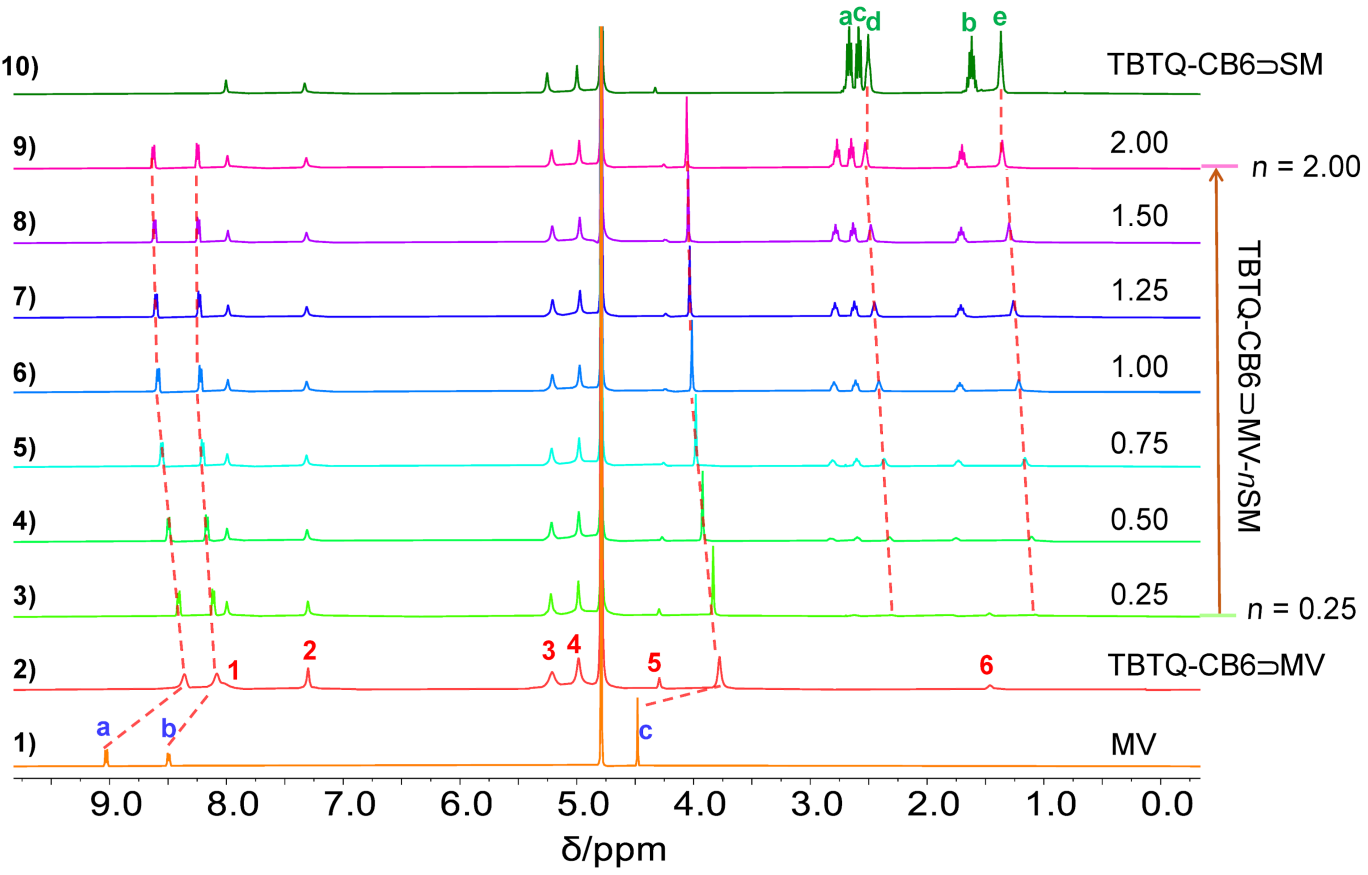
^1^H NMR spectra (400 MHz, D_2_O, 25 °C) of (1) **MV** (3 mM), (2) **TBTQ-CB6** and **MV** (3 mM each), (3–9) **TBTQ-CB6** and **MV** (3 mM each) with gradual addition of **SM**, and (10) **TBTQ-CB6** and **SM** (3.0 mM each).

**Competitive replacement of DOX from the TBTQ-CB6**
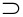
**DOX complex by SM.** Similarly, ^1^H NMR titration experiments were performed to determine the competing effect of **SM** and the release of **DOX** from the **TBTQ-CB6**
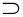
**DOX** complex. **SM** was added to **TBTQ-CB6**
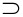
**DOX** (3.0 mM) in amounts ranging from 0.25 to 2.00 equivalents. As shown in Figure 6, the proton signals of **DOX** were strongly broadened in **TBTQ-CB6**
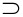
**DOX** and after the addition of **SM**. Nevertheless, it can be observed that the broadened H_f_ of **DOX** reappears as a small doublet after the addition of 1.00 equivalent of **SM**. Moreover, the proton signals (H^1^, H^2^, and H^3^) of the host **TBTQ-CB6** first became sharpened and then broadened again with the addition of 0.25 to 1.00 equivalents of **SM**, and the signals remained essentially unchanged thereafter. These observations suggest that **DOX** was successfully released from the cavity of the host **TBTQ-CB6**. Furthermore, the resonances of the protons H_d_ and H_e_ of **SM** were located upfield as compared to those of the free **SM** (see Figure 3) and the **TBTQ-CB6**
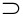
**SM** complex, which suggests that **SM** occupied the cavity of **TBTQ-CB6**. These results indicate that **DOX** was effectively released from the **TBTQ-CB6**
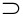
**DOX** complex by competitive binding of **SM** to **TBTQ-CB6** due to the approximately 7.5-fold higher binding affinity of **TBTQ-CB6**
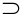
**SM** than **TBTQ-CB6**
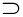
**DOX**.

**Figure 6:**
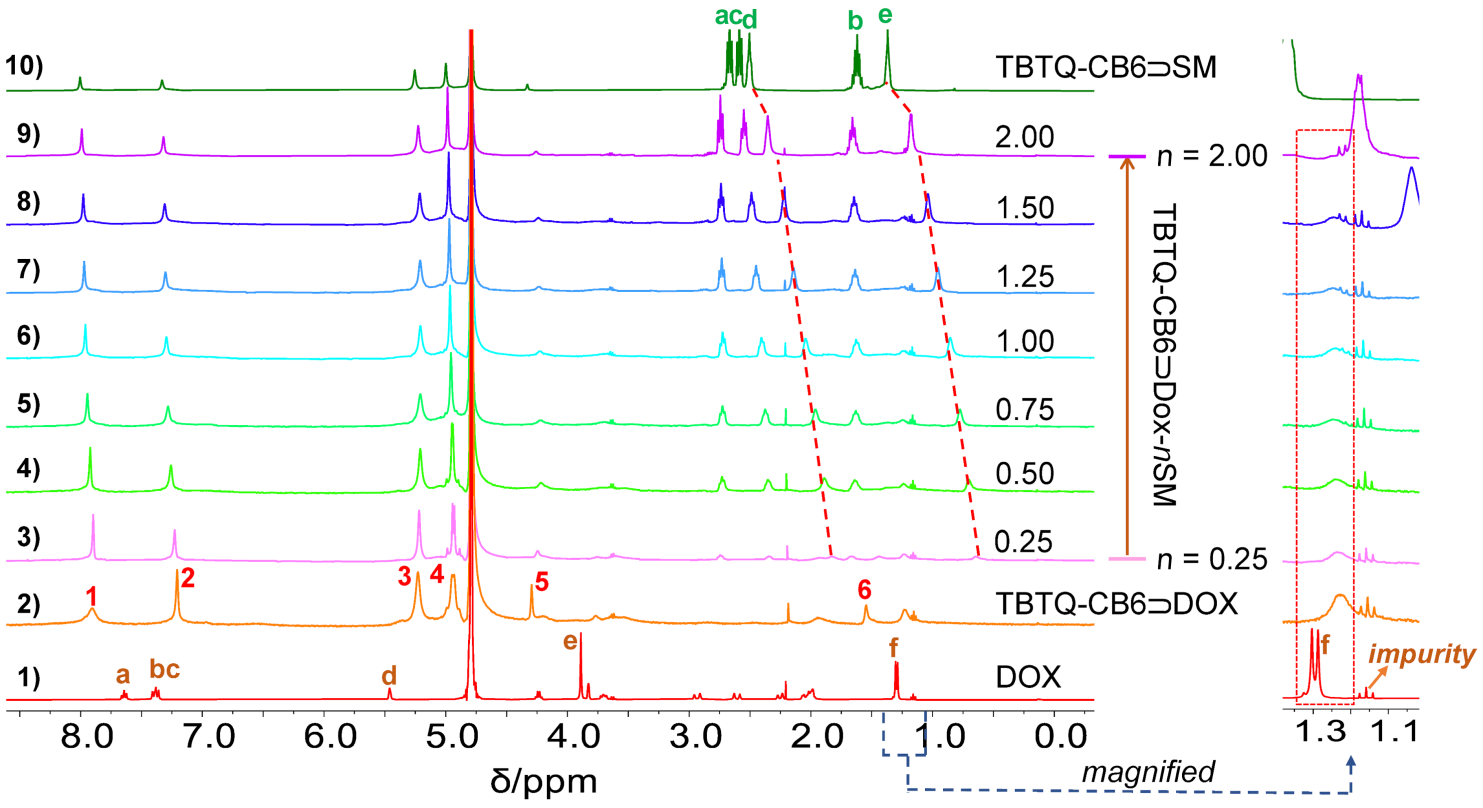
^1^H NMR spectra (400 MHz, D_2_O, 25 °C) of (1) **DOX** (3 mM), (2) **TBTQ-CB6** and **DOX** (3 mM each), (3–9) **TBTQ-CB6** and **DOX** (3 mM each) with gradual addition of **SM**, (10) **TBTQ-CB6** and **SM** (3.0 mM each).

## Conclusion

In conclusion, we designed and synthesized a novel water-soluble tribenzotriquinacene receptor, **TBTQ-CB6**, as a molecular-scale drug carrier for encapsulating the anticancer drugs dimethyl viologen (**MV**) and doxorubicin (**DOX**) via host–guest interactions. The target compound **TBTQ-CB6** was characterized in detail by NMR spectroscopy and mass spectrometry. The host–guest complexes of **TBTQ-CB6** with **MV**, **DOX**, and the overexpressed molecule spermine (**SM**) found within cancer cells are formed in a 1:1 stoichiometry in all cases and with association constants of *K*_a_ = (7.67 ± 0.34) × 10^4^ M^−1^, *K*_a_ = (6.81 ± 0.33) × 10^4^ M^−1^, and *K*_a_ = (5.09 ± 0.98) × 10^5^ M^−1^, respectively. As a result of the higher binding affinity between **TBTQ-CB6** and **SM**, **MV** and **DOX** are released from their corresponding host–guest complexes by the competitive replacement effect of **SM**, as confirmed by ^1^H NMR titration experiments. This host–guest competitive substitution system may have potential applications in controlled drug delivery.

## Experimental

**General information.** All commercially available reagents were used as received unless otherwise specified. Anhydrous solvents were collected from a Mikrouna Solv Purer G3 solvent purification system. The ^1^H NMR and ^13^C NMR spectra were recorded on a 400 MHz Bruker NMR spectrometer and chemical shifts are reported in ppm (δ). The fluorescence spectra were measured on a HORIBA Fluorolog-3 spectrofluorometer. Mass spectra were recorded using either the ultrafleXtreme MALDI-TOF mass spectrometer (Bruker) with α-cyano-4-hydroxycinnamic acid (HCCA) as a matrix, or by electrospray ionization (ESI) on a Waters G2-XS QTOF instrument connected to a Waters H-class UPLC equipped with a Waters BEH C18 column using an eluent consisting of 90% acetonitrile with 0.1% formic acid and 10% water with 0.1% formic acid at a flow rate of 0.4 mL/min. Freeze-drying was conducted on a Scientz-18N freeze-dryer.

**Synthesis of compound 2.** A mixture of compound **1** (2.30 g, 3.7 mmol), ethyl azidoacetate (5.76 g, 44.7 mmol), copper(II) sulfate pentahydrate (0.93 g, 3.7 mmol) and sodium ascorbate (1.48 g, 7.4 mmol) in a cosolvent of tetrahydrofuran/water (60 mL/30 mL) was stirred vigorously under nitrogen atmosphere at 60 °C for 24 h. After the solvent was removed by rotary evaporation, a 0.1 M aqueous solution of EDTA (20 mL) was added and the mixture was further stirred for 1 h. Following the addition of water (100 mL), the mixture was extracted with dichloromethane (3 × 50 mL). A substantial amount of solvent was removed from the combined organic layers to produce a concentrated solution. Afterward, diethyl ether was added and the precipitate was collected by suction filtration, washed, and dried to obtain compound **2** as a yellow solid (3.76 g, 73%). Mp 104.8–106.6 °C; ^1^H NMR (400 MHz, DMSO-*d*_6_, 25 °C) δ 8.18 (s, 6H), 7.36 (s, 6H), 5.36 (s, 12H), 5.21, 5.17 (ABq, *J* = 12.4 Hz, 12H), 4.21 (s, 3H), 4.15 (q, *J* = 7.1 Hz, 12H), 1.58 (s, 3H), 1.19 (t, *J* = 7.1 Hz, 18H); ^13^C NMR (100 MHz, DMSO-*d*_6_, 25 °C) δ 167.20, 147.89, 143.15, 137.57, 126.07, 110.42, 62.61, 62.55, 62.15, 61.50, 50.38, 27.48, 13.93; (+)-ESI-HRMS (*m*/*z*): [M + H]^+^ calcd for C_65_H_73_N_18_O_18_, 1393.5345; found, 1393.5354 (Δ = +0.7 ppm).

**Synthesis of compound 3.** To a methanol solution (10 mL) of compound **2** (0.50 g, 0.36 mmol), 40% aqueous sodium hydroxide was added. The mixture was heated under reflux for 12 h. The solvent was removed by distillation and the residue was dissolved in water and the pH of the solution was adjusted to 2 with hydrochloric acid. The resulting precipitate was collected by suction filtration, and washed sequentially with water, dichloromethane, and acetone to give compound **3** as a colorless solid (0.37 g, 85%). Mp 59.6–60.7 °C; ^1^H NMR (400 MHz, DMSO-*d*_6_, 25 °C) δ 8.19 (br s, 6H), 7.38 (br s, 6H), 5.26 (br s, 12H), 5.21 (br s, 12H), 4.23 (s, 3H), 1.60 (s, 3H); ^13^C NMR (100 MHz, DMSO-*d*_6_, 25 °C) δ 168.55, 148.01, 143.23, 137.64, 126.25, 110.44, 62.71, 62.63, 62.26, 50.89, 27.58; MALDI-TOF MS (*m*/*z*) [M + H]^+^ calcd for C_53_H_49_N_18_O_18_, 1225.3467; found, 1225.3449 (Δ = −1.5 ppm).

**Synthesis of compound TBTQ-CB6.** Compound **3** (0.27 g, 0.22 mmol) was dissolved in a 40% ammonium hydroxide solution (30 mL) and stirred at room temperature for 5 h. The solvent was removed by distillation, and the residue was dissolved in water and washed several times with dichloromethane. The aqueous layer was collected and freeze-dried to afford compound **TBTQ-CB6** as a colorless solid (0.28 g, 97%). Mp 198.2–199.4 °C; ^1^H NMR (400 MHz, D_2_O, 25 °C) δ 7.89 (s, 6H), 7.21 (s, 6H), 5.25 (s, 12H), 4.97, 4.91 (ABq, *J* = 17.2 Hz, 12H), 4.31 (s, 3H), 1.57 (s, 3H); ^13^C NMR (100 MHz, D_2_O, 25 °C) δ 172.58, 147.37, 143.07, 138.51, 126.22, 110.74, 63.11, 62.15, 61.82, 52.77, 24.69; MALDI-TOF MS (*m*/*z*) [M – 6NH_4_ + 6H + Na]^+^ calcd for C_53_H_48_N_18_O_18_Na, 1247.3286; found, 1247.3211 (Δ = −6.0 ppm); [M – 6NH_4_ + 7H]^+^ calcd for C_53_H_49_N_18_O_18_, 1225.3467; found, 1225.3252 (Δ = −17.6 ppm), and [M – 6NH_4_ + 5H + 2 Na]^+^ calcd for C_53_H_47_N_18_Na_2_O_18_, 1269.3106; found, 1269.3199 (Δ = + 7.3 ppm).

## Supporting Information

File 1 Copies of NMR and mass spectra and fluorescence spectra of **TBTQ**–**CB6** with **MV** and **DOX**, respectively, at different ratios.
