# Supplementary material for: Synthesis of a new water-soluble hexacarboxylated tribenzotriquinacene derivative and its competitive host–guest interaction for drug delivery
Source: Beilstein J Org Chem. 2022 May 12;18:539–48. doi: 10.3762/bjoc.18.56 (PMC9112186; doi:10.3762/bjoc.18.56)
Supplement: File 1 — Copies of NMR and mass spectra and fluorescence spectra of TBTQ–CB6 with MV and DOX, respectively, at different ratios. [file Beilstein_J_Org_Chem-18-539-s001.pdf]

BEILSTEIN JOURNAL OF ORGANIC CHEMISTRY

Supporting Information

for

Synthesis of a new water-soluble hexacarboxylated tribenzotriquinacene derivative and its competitive host–guest interaction for drug delivery

Man-Ping Li, Nan Yang and Wen-Rong Xu

Beilstein J. Org. Chem. **2022,** *18,* 539–548. doi:10.3762/bjoc.18.56

Copies of NMR and mass spectra and fluorescence spectra of TBTQ–CB6 with MV and DOX, respectively, at different ratios

License and Terms: This is a supporting information file under the terms of the Creative Commons Attribution License (https://creativecommons.org/licenses/by/4.0). Please note that the reuse, redistribution and reproduction in particular requires that the author(s) and source are credited and that individual graphics may be subject to special legal provisions.
The license is subject to the *Beilstein Journal of Organic Chemistry* terms and conditions: (https://www.beilstein-journals.org/bjoc/terms)

Table of contents

Figure S1: ^{1}H NMR spectrum of compound **2** S1

Figure S2: ^{13}C NMR spectrum of compound **2**.. S1

Figure S3: (+)-ESI mass spectrum of compound **2** S2

Figure S4: ^{1}H NMR spectrum of compound **3** S2

Figure S5: ^{13}C NMR spectrum of compound **3** S3

Figure S6: MALDI-TOF mass spectrum of compound **3**.. S3

Figure S7: ^{1}H NMR spectrum of compound **TBTQ-CB6** S4

Figure S8: ^{13}C NMR spectrum of compound **TBTQ-CB6** S4

Figure S9: MALDI-TOF mass spectrum of compound **TBTQ-CB6**................................. S5

Figure S10: Fluorescence spectra of the mixture of **TBTQ-CB6** and **MV** in different molar ratios at a constant total concentration of 10 μM S5

Figure S11: Fluorescence spectra of the mixture of **TBTQ-CB6** and **DOX** in different molar ratios at a constant total concentration of 10 μM S6

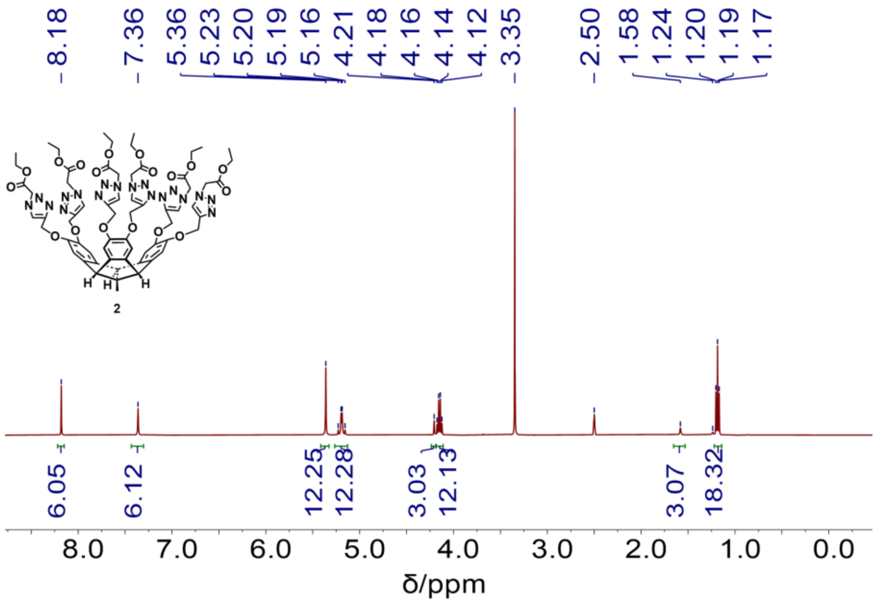

Figure S1: ^{1}H NMR spectrum of compound **2** (400 MHz, DMSO-d_6, 25 °C).

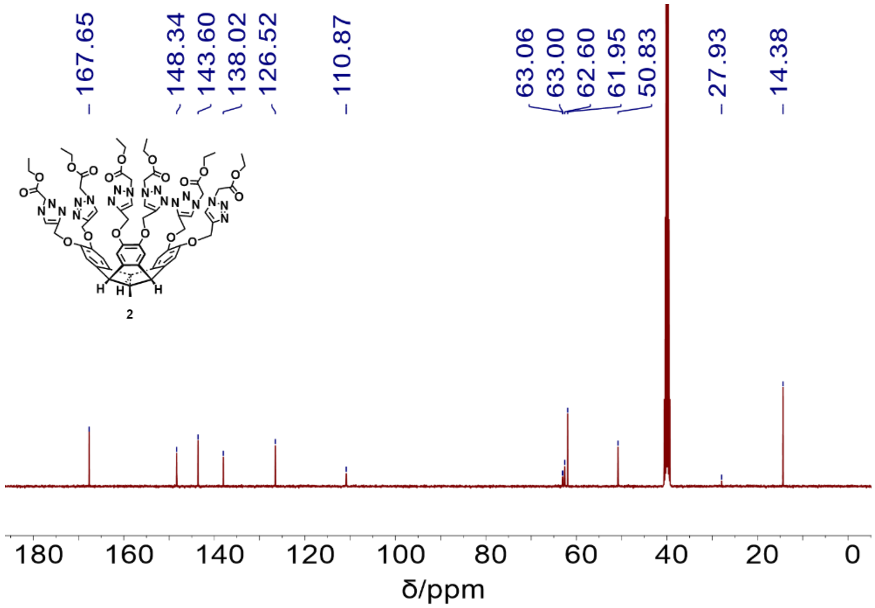

Figure S2: ^{13}C NMR spectrum of compound **2** (100 MHz, DMSO-d_6, 25 °C).

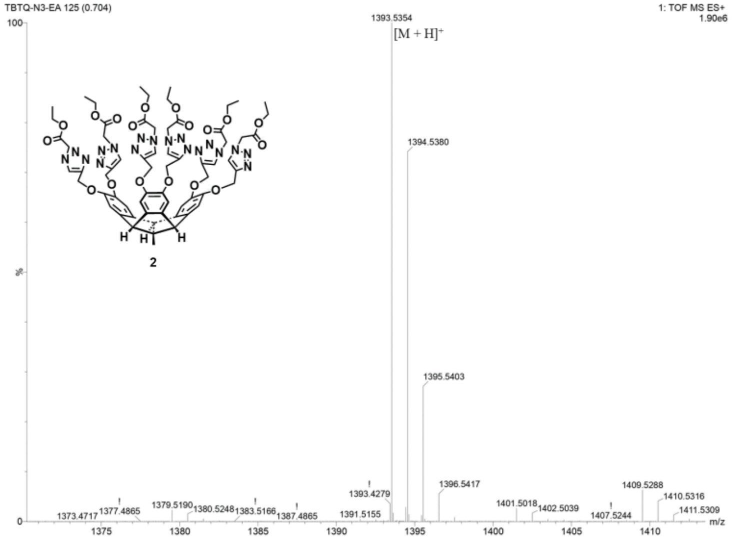

Figure S3: (+)-ESI mass spectrum of compound **2**.

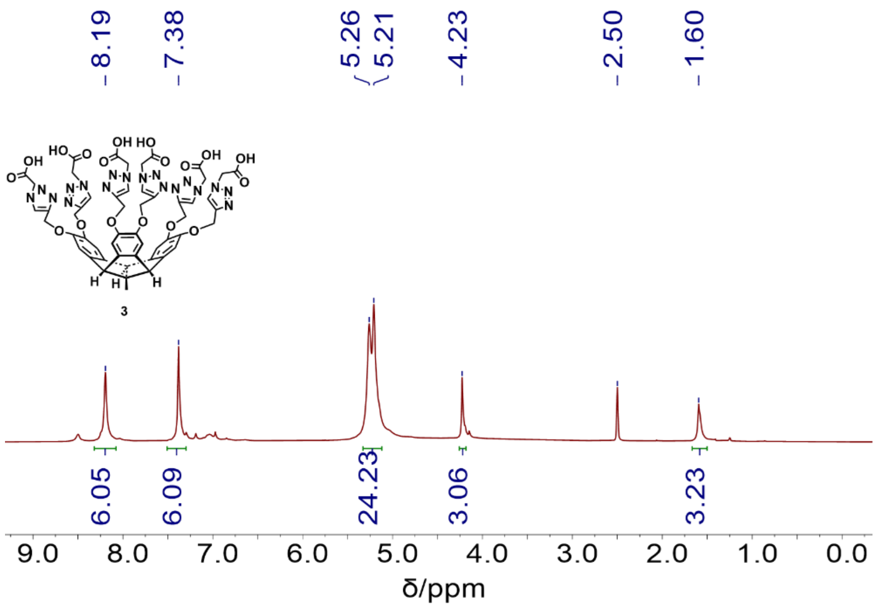

Figure S4: ^{1}H NMR spectrum of compound **3** (400 MHz, DMSO-d_6, 25 °C).

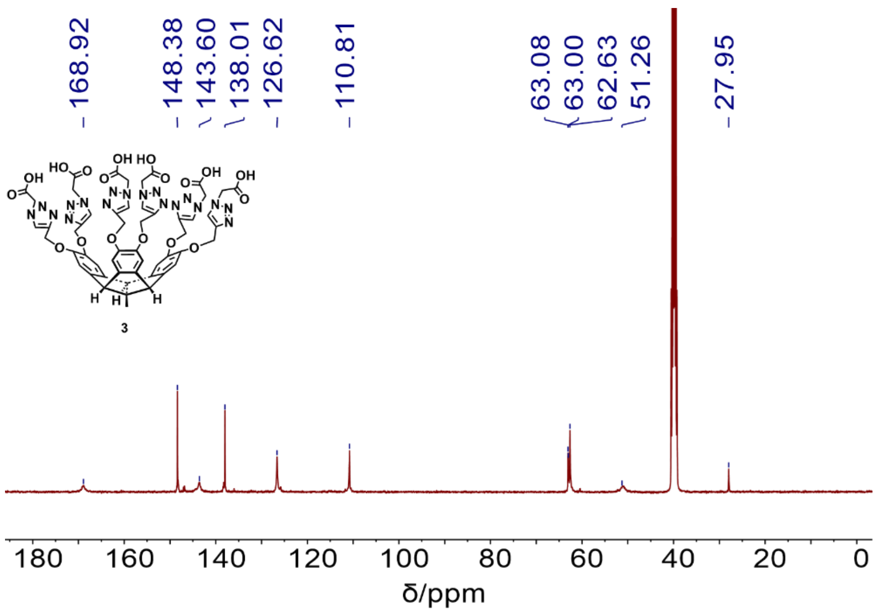

Figure S5: ^{13}C NMR spectrum of compound **3** (100 MHz, DMSO-d_6, 25 °C).

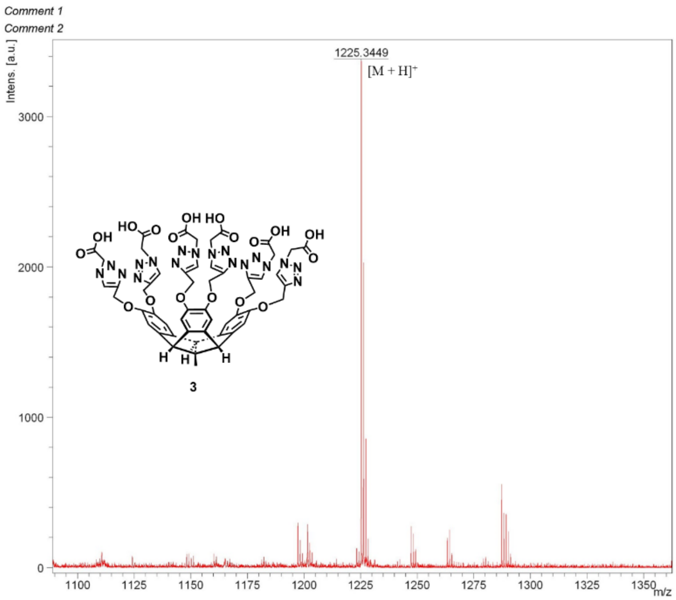

Figure S6: MALDI-TOF mass spectrum of compound **3**.

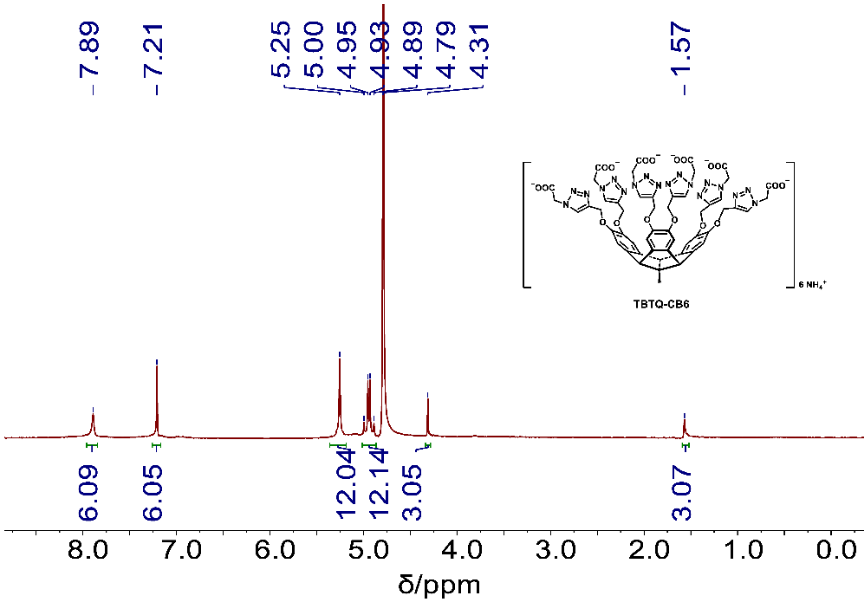

Figure S7: ^{1}H NMR spectrum of compound **TBTQ-CB6** (400 MHz, D_2O, 25 °C).

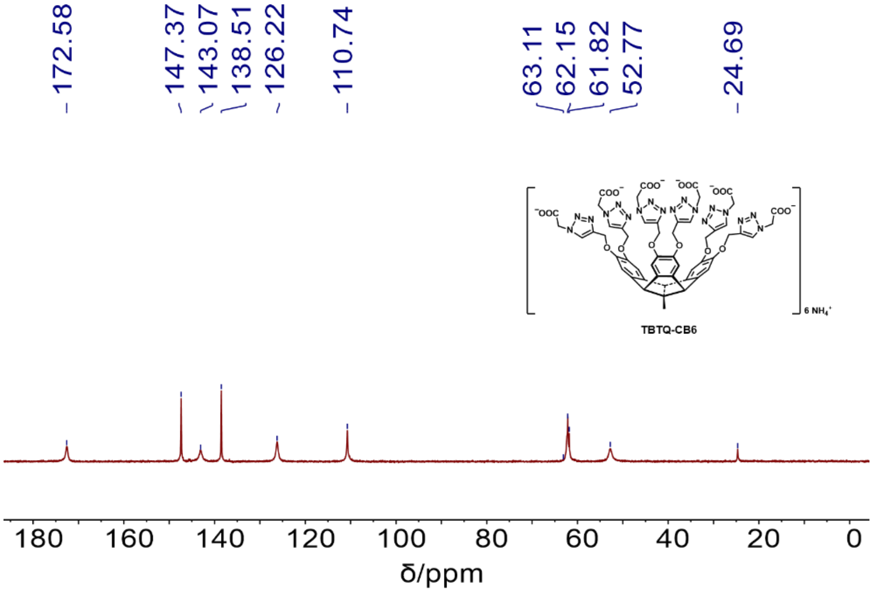

Figure S8: ^{13}C NMR spectrum of compound **TBTQ-CB6** (100 MHz, D_2O, 25 °C).

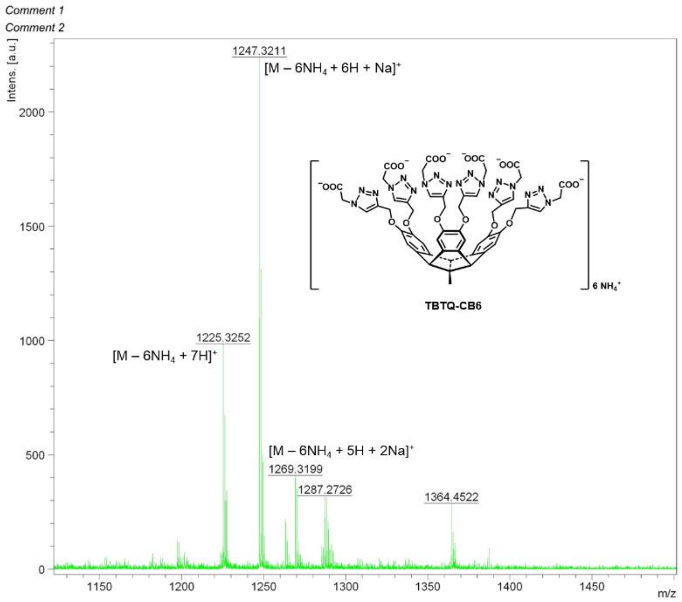

Figure S9: MALDI-TOF mass spectrum of compound **TBTQ-CB6**.

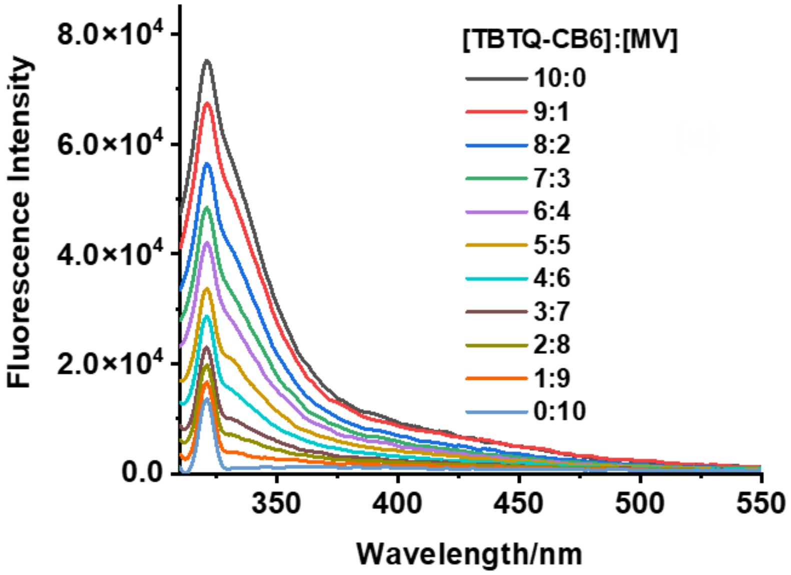

Figure S10: Fluorescence spectra of the mixture of **TBTQ-CB6** and **MV** in different molar ratios at a constant total concentration of 10 μM.

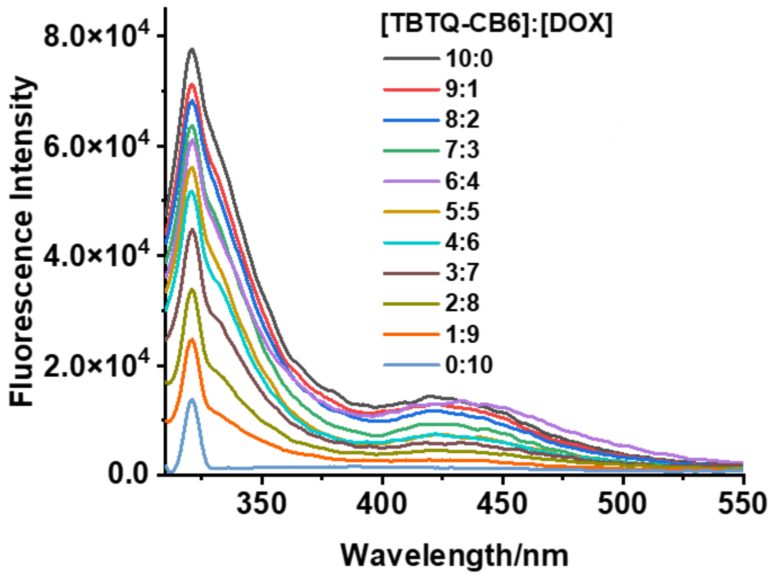

Figure S11: Fluorescence spectra of the mixture of **TBTQ-CB6** and **DOX** in different molar ratios at a constant total concentration of 10 μM.
